# Atlas of craniovertebral junction surgery- problems and solutions

**DOI:** 10.4103/0972-2327.48873

**Published:** 2009

**Authors:** Ravi Mohan Rao

**Affiliations:** Professor, Neurosurgery, SCTIMST, Trivandrum, India

Atlas of craniovertebral (CV) junction surgery by Dr. Hillol K Pal is a novel way of presenting intricacies of an obviously difficult area for most neurosurgeons. The atlas is a collection of cases tackled by the author, which has been presented as individual case reports. These case reports have been classified into six sections, namely, congenital anomalies, traumatic lesions, tuberculosis of the CV Junction, neoplastic disorders, vascular lesions, and degenerative disorders. Every problem occurring in CV junction has been covered in an exhaustive manner, which includes clinical presentation, investigations, solutions offered by the author for each problem followed by literature review and detailed references. Each case report has been backed by superb illustrations of X-rays, MRI scans, CT scan, and high-resolution operative photomicrographs. The layout has been carefully planned to enhance the high reference value of the book. The scientific contents with evidences and other important information provided in this book are of high relevance and undoubtedly makes this a must read for the young neurologists/neurosurgeons.

Although there are many high-quality monograms, atlases on the same topic authored by many well-known neurosurgeons, the presentation in this book is different because it is a compilation of case reports. Therefore, the beginner will have to go through an entire section to glean information that is available in a single chapter in other books. The main drawback is that the author's reasoning for choosing a particular surgical approach is not clear for a beginner.

On the whole, the book is refreshingly different and is worth keeping in the library as a reference book.

